# TRIM52 promotes colorectal cancer cell proliferation through the STAT3 signaling

**DOI:** 10.1186/s12935-019-0775-4

**Published:** 2019-03-14

**Authors:** Shengli Pan, Yingying Deng, Jun Fu, Yuhao Zhang, Zhijin Zhang, Xiaokun Ru, Xianju Qin

**Affiliations:** 1 Division of Gastrointestinal Surgery, Department of General Surgery, Shanghai Eighth People’s Hospital, No. 8 Caobao Road, Xuhui District, Shanghai, 200232 China; 2 Department of Ophtalmology, Shanghai Eighth People’s Hospital, Shanghai, China

**Keywords:** TRIM52, Proliferation, Prognosis, STAT3

## Abstract

**Background:**

The tripartite motif (TRIM) family proteins are implicated in the pathogenesis of various human malignancies. The up-regulation and oncogenic roles of TRIM52 have been reported in hepatocellular carcinoma. In the current study, we aimed to examine its expression and possible function in colorectal cancer (CRC).

**Method:**

Immunohistochemical staining or immunoblotting analysis was carried out to detect protein expression. Cell proliferation and apoptosis was evaluated by Cell Counting Kit-8 (CCK-8) and flow cytometry assay, respectively.

**Results:**

TRIM52 expression was increased in 67.5% of CRC tissues (54/80) compared to matched normal colonic mucosa. TRIM52 expression was closely related with tumor size (*p *= 0.0376), tumor stage (*p *= 0.0227) and overall survival (*p *= 0.0177). Short hairpin RNAs (shRNAs) targeting TRIM52 had the potential anti-proliferative effects on CRC cell lines, SW480 and LoVo, by inducing cell apoptosis. In addition, an in vivo xenograft experiment confirmed the in vitro results. In addition, TRIM52 shRNAs decreased the phosphorylation of STAT3, but increased the protein expression of SHP2, a negative regulator of STAT3 phosphorylation. TRIM52 formed a complex with SHP2 and promoted the ubiquitination of SHP2. Furthermore, inhibition of the STAT3 signaling by AG490 in RKO cells significantly abolished the effects of TRIM52 overexpression on cell proliferation, apoptosis and STAT3 activation.

**Conclusions:**

TRIM52 might exert oncogenic role in CRC via regulating the STAT3 signaling pathway.

**Electronic supplementary material:**

The online version of this article (10.1186/s12935-019-0775-4) contains supplementary material, which is available to authorized users.

## Background

Colorectal cancer (CRC) is the third most common form of gastrointestinal cancers, with more than one million newly diagnosed cases each year in the world [1]. The incidence of CRC in women and men is 9.2% and 10%, respectively, which makes CRC the second most common among women, and the third most common cancer among men [2–4]. Although continued efforts have made to improve the understanding of tumorigenesis, and numerous oncogenes and tumor suppressor genes involved in CRC tumorigenesis have been identified, the biological and molecular mechanisms of CRC are still far from being fully understood.

Signal transducer and activator of transcription 3 (STAT3), a transcription factor, normally resides in the cytoplasm. When its tyrosine-705 residue is phosphorylated in response to cytokine signaling and tyrosine kinase oncoproteins, STAT3 translocates to the nucleus and controls the transcription of downstream genes that are involved in cell cycle transition and cell survival [5, 6]. Activated phospho-STAT3 (p-STAT3) is found to be increased in CRC samples compared to normal mucosae [7–9]. The Janus family kinases (JAK) are known to mediate the activation of STAT3 [10]. AG490, a pharmacological inhibitor of JAK, and STAT3 small interfering RNA (siRNA) could suppress CRC cell growth and invasion, and induce CRC cell apoptosis [9].

The tripartite motif (TRIM) family protein is characterized by its tripartite motif, which contains a RING domain, one or two B-box domains and a coiled-coil domain [11]. More than 80 members have been identified in human until now. Substantial evidence has accumulated and supported the roles of TRIM proteins in innate immune response, cell proliferation, apoptosis and autophagy [11, 12]. The dysfunction of TRIM family proteins is known to be associated with the pathogenesis of several diseases, including various human cancers [12–14]. TRIM52 is a novel member of the TRIM protein family [15]. TRIM52 can degrade the viral nonstructural protein 2A (NS2A), thus exerting antiviral activity against Japanese encephalitis virus (JEV) infection [16]. TRIM52 is able to active the NF-κB signaling pathway [17]. Recently, the oncogenic role of TRIM52 has been described in hepatocellular carcinoma (HCC) [18, 19]. Hepatitis B virus X protein (HBx) may regulate TRIM52 expression [19]. TRIM52 can facilitate cell proliferation, migration and invasion of HCC cells [18].

Previous studies have revealed the significance of TRIM52 in HCC, but no investigation has focused on the effects of TRIM52 on CRC. In the current study, we examined the expression and function of TRIM52 in CRC. We found that the STAT3 signaling was involved in the abilities of TRIM52 to induce the proliferation and inhibit apoptosis in CRC cells.

## Materials and methods

### Tissue collection

This study included 80 patients with CRC who underwent surgery at the Department of Gastrointestinal Surgery, Shanghai Eighth People’s Hospital from January 2008 to December 2011. Clinical pathology features, including gender, age, tumor stage, tumor size, and histological type, were retrieved from the medical records (Table 1). The mean age of the enrolled patients ranged from 58 years (34–75 years), including 43 males and 37 females. Collected CRC tissues (n = 80) and adjacent normal colonic mucosa were formalin-fixed paraffin-embedded. This study was approved by the Ethics Review Committee of Shanghai Eighth People’s Hospital (Shanghai, China). All participants received written informed consent.

**Table 1:** Clinicopathological characteristics and TRIM52 expression (n = 80)

Characteristic	Cases	%
*Gender*		
Male	43	53.8
Female	37	46.2
*Age (years)*		
≥ 65	47	58.8
< 65	33	41.2
*Tumor size (cm)*		
≥ 5.0	45	56.3
< 5.0	35	43.7
*Clinical stage*		
I/II	37	46.3
III	43	53.7
*Histological types*		
Non-mucinous adenocarcinoma	60	75.0
Mucinous adenocarcinoma	20	25.0
*TRIM52 expression*		
Low	32	40.0
High	48	60.0
*Vital status (at followed-up)*		
Alive	20	25.0
Dead	60	75.0

### Immunohistochemistry (IHC) analysis

For IHC analyses, formalin-fixed paraffin-embedded tissue samples were cut into 5 μm-thick section. Antigen retrieval was performed in 0.01 M citrate sodium buffer solution (pH = 6.0) at 100 °C for 10 min. Endogenous peroxidase was blocked in 0.3% H_2_O_2_ at room temperature for 15 min. Non-specific binding was blocked by incubation in 10% bovine serum albumin 10% for 30 min. Then, a rabbit primary antibody against TRIM52 (diluted in 1:50; Novus Biologicals, Inc., Littleton, CO, USA; NBP2-31651) was added for overnight incubation at 4 °C. After washed three times in phosphate-buffered saline, anti-rabbit IgG was applied for 1 h incubation at room temperature. The sections were developed with DAB substrate and counterstained with hematoxylin. The stained sections were evaluated by two investigators, and classified into the TRIM52-high expression group and the TRIM52-low expression group with 20% of tumor cell positive stained as cut-off.

### Cell culture

Five human CRC cell lines (SW480, LoVo, SW620, HT29 and RKO) and normal human intestinal crypt cells (HIEC) were purchased from the Cell Bank of the Chinese Academy of Sciences (Shanghai, China). All the cells were grown in DMEM medium (Invitrogen, Carlsbad, CA, USA) containing 10% fetal bovine serum (Invitrogen) and 1% penicillin/streptomycin in a humidified air with 5% CO_2_ at 37 °C.

### Lentiviral construction

Four TRIM52-specific target sequences were designed using Dharmacon siDESIGN Center (Dharmacon, Waltham, USA). Short hairpin RNAs (shRNAs) targeting these sequences (RNAi#1, RNAi#2, RNAi#3 and RNAi#4) and a negative control sequence (NC) were synthesized (Additional file 1: Table S1) by Genechem (Shanghai, China), and constructed into pLKO.1 vector (Addgene, Cambridge, MA, USA). The coding sequence of TRIM52 was amplified by reverse transcription PCR with the following primers (forward 5′-CGGAATTCATGGCTGGTTATGCCACTACTC-3′ and reverse 5′-CGGGATCCTTACTGATTATAGGCCTTGCTG-3′) and constructed into pLVX-puro vector (Clontech, Palo Alto, CA, USA). TRIM52 shRNAs lentiviruses, TRIM52 overexpression lentivirus (TRIM52OE), and control (NC and Vector) lentiviruses were produced by transient transfection of lentiviral constructs and packaging vectors into 293T cells with Lipofectamine 2000 (Invitrogen).

### Immunoblot assay

Total protein was extracted by radio-immunoprecipitation assay (RIPA) buffer containing phosphatase and protease inhibitors (Beyotime, Shanghai, China). Equal amounts of protein from each sample were separated by 10% or 12% sodium dodecyl sulfatepolyacrylamide gel electrophoresis (SDS-PAGE), and then electro-transferred onto nitrocellulose membranes (Millipore, Bredford, MA, USA). Following blocking with 5% skim milk in Tris-buffered saline Tween-20 (TBST) for 30 min at room temperature, the membranes were probed with the following primary antibodies: TRIM52 antibody from San Cruz (Santa Cruz, CA, USA), Bcl-2, Bax, p-STAT3, STAT3, PTP1B, TCPTP, SHP1, SHP2 and ubiquitin (Ub) antibodies from Abcam (Cambridge, MA, USA), and GAPDH (glyceraldehyde-3-phosphate dehydrogenase) antibody from Cell Signaling Technology (Danvers, MA, USA) according to the manufacturers’ protocols. After being rinsed by TBST, the membranes were incubated with incubated with peroxidase labeled secondary antibody at room temperature for 1 h. Signal was detected with enhanced chemiluminescence substrate (ECL, BioRad, Richmond, CA, USA).

### Immunoprecipitation

Total protein was incubated with protein A/G Plus agarose beads (Santa Cruz Biotech., Santa Cruz, CA, USA) together with specific antibodies. After incubation for 4 h at 4 °C, protein A/G Plus beads were washed three times with RIPA buffer. Immunoprecipitates were mixed with SDS-PAGE loading buffer and boiled at 95 °C for 5 min. The immunoprecipitates were analyzed by western blotting with antibodies against TRIM52, SHP2 or ubiquitin (Ub).

### Cell Counting Kit-8 (CCK-8) assay

Cells incubated in 96-well plates were treated as indicated and cell proliferation was assessed by CCK-8 assay (SAB biotech. College Park, MD, USA) at 0, 24, 48 and 72 h post treatment following the manufacturer’s instruction. Optical density (OD) was recorded at 450 nm.

### Flow cytometry

At 48 h post treatment, cell apoptosis was assessed by the Annexin V-FITC/PI apoptosis detection kit (Beyotime) and BD Biosciences Accuri C6 flow cytometer (Franklin Lakes, NJ, USA) following the manual procedure.

### Tumor xenograft implantation in nude mice

The animal study followed the Guidelines for the Animal Care and Use approved by Shanghai Eighth’s People Hospital. Nude mice, aged 5–6 weeks old, were housed in a specific pathogen free (SPF) grade laboratory with a constant temperature (22–25 °C) and humidity (55 ± 5%). A total of 12 mice were allocated into RNAi#1 group and NC group with 6 mice in each group. LoVo cells stably expressing RNAi#1 or NC were established by puromycin selection. The stable cells were resuspended in serum-free DMEM were injected into each mouse (10^6^ cells per mouse). Tumor volume was calculated with the following formula: volume = 1/2 × (largest diameter) × (smallest diameter)^2^ every 3 days after inoculation. After 33 days, the nude mice were sacrificed, and the xenografts were collected, weighed and processed for TUNEL (Terminal deoxynucleotidyl transferase [TdT]-mediated deoxyuridine triphosphate (dUTP)-nick end labeling) staining (Roche, Indianapolis, IN, USA).

### Statistical analysis

Data analysis was performed with GraphPad Prism software (GraphPad Software, La Jolla, CA, USA). Fisher’s exact test was conducted to analyze the relationship between TRIM52 expression and clinical features. Kaplan–Meier survival curves followed by log-rank test were used to compare overall survival of different groups. The in vitro experiments were repeated at least three times. Student’s *t* test and one-way analysis of variance (ANOVA) followed by Tukey’s multiple comparison were carried out for comparison of two groups and for comparison of three or more groups, respectively. *p *< 0.05 was considered significant.

## Results

### TRIM52 protein expression is up-regulated in human CRC tissues

**Fig. 1:**
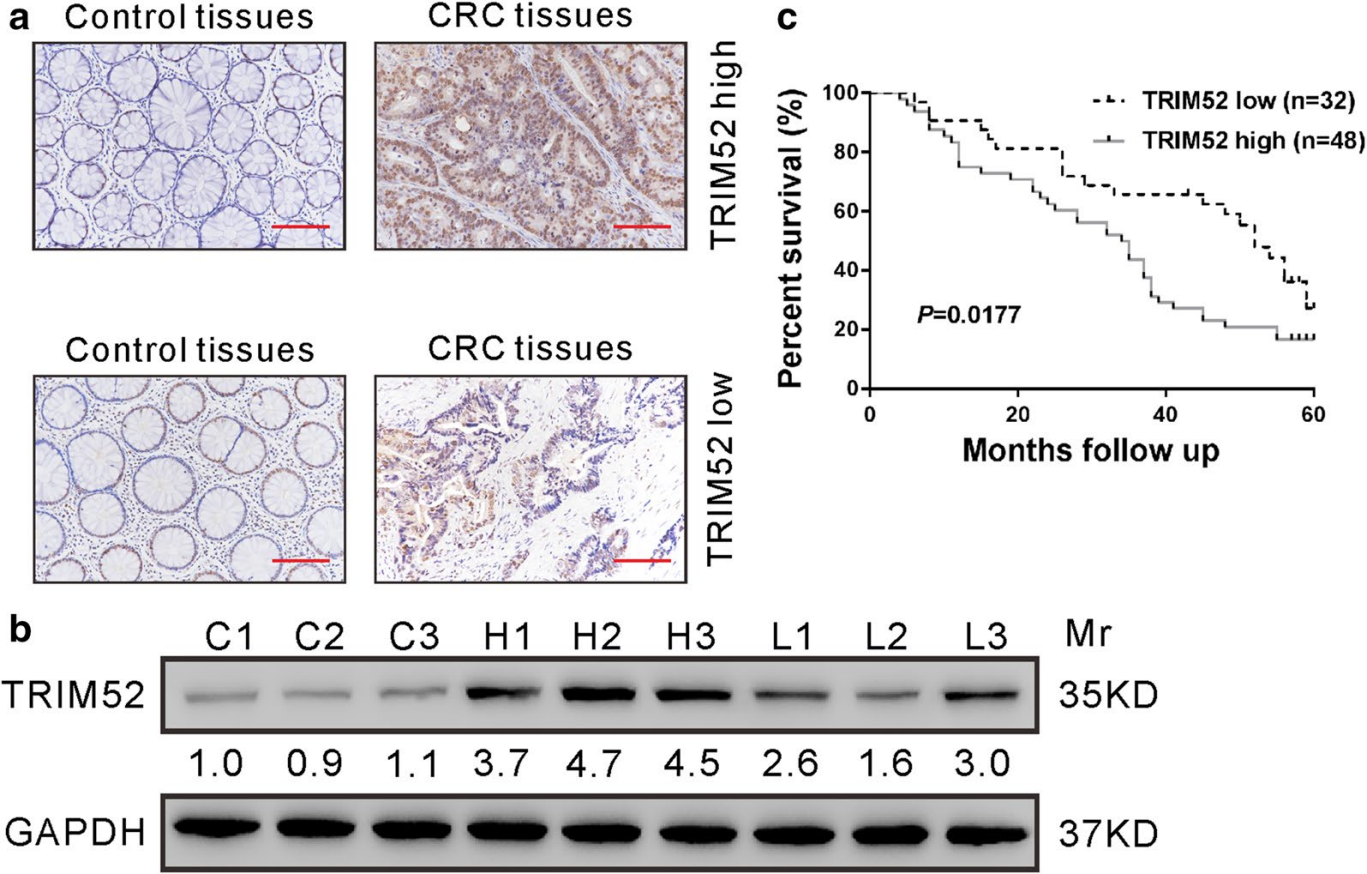
Increased expression of TRIM52 in human CRC tissues. **a** IHC analysis showed that TRIM52 expression was significantly up-regulated and down-regulated in 48 and 32 cases of CRC tissues, respectively. Representative images are shown. Scale bar: 100 μm. **b** Western blotting analysis was performed on 3 normal colonic mucosa specimens (C1–C3), 3 CRC specimens with up-regulated expression of TRIM52 (H1–H3) and 3 CRC specimens with down-regulated expression of TRIM52 (L1–L3). The relative band density was obtained using ImageJ software (http://rsb.info.nih.gov/ij/, Bethesda, MD, USA) with GAPDH as loading control and shown below the blot. **c** Kaplan–Meier survival curves showed a significant difference in overall survival between patients with high or low expression of TRIM52

To examine TRIM52 expression in CRC tissues, IHC staining was performed in archived paraffin CRC specimens and paired normal colonic mucosa specimens from 80 patients. We found that TRIM52 expression was significantly up-regulated in 67.5% CRC tissues (54/80) compared to matched normal colonic mucosa (Fig. 1a) Western blotting analysis on 3 normal colonic mucosa specimens (C1–C3), 3 CRC specimens from up-regulated group and 3 CRC specimens from down-regulated group (L1–L3) validated the IHC results (Fig. 1b).

#### Increased TRIM52 expression is correlated with the poor prognosis of CRC patients

Next, we estimated the correlation between TRIM52 expression and clinicopathologic features of CRC patients. The patients were categorized into two groups, TRIM52 low group (n = 32) and TRIM52 high group (n = 48), based on the positive staining ratio of TRIM52 in cancer cellsBy Fisher’s exact test, we found that TRIM52 levels were significantly correlated with tumor size (*p *= 0.0376) and tumor stage (*p *= 0.0227) (Table 2). Although TRIM52 levels did not show a statistically significant correlation with vital status (at followed-up) (*p *= 0.0633), Kaplan–Meier and log-rank survival analysis showed a significant correlation between high expression of TRIM52 and poor overall survival of patients with CRC (*p *= 0.0177, Fig. 1c).

**Table 2:** Correlation of TRIM52 expression in colorectal cancer tissues with different clinicopathological features (n = 80)

Characteristic	TRIM52		*P*-value
	Low (n = 32)	High (n = 48)	
*Gender*			0.6504
Male	16	27	
Female	16	21	
*Age (years)*			0.4888
≥ 65	17	30	
< 65	15	18	
*Tumor size (cm)*			0.0376*
≥ 5.0	13	32	
< 5.0	19	16	
*Clinical stage*			0.0227**
I/II	20	17	
III	12	31	
*Histological types*			0.3061
Non-mucinous adenocarcinoma	22	38	
Mucinous adenocarcinoma	10	10	
*Vital status (at followed-up)*			0.0633
Alive	12	8	
Dead	20	40	

Clinicopathological features were assessed using the Fisher’s exact test

* *p *< 0.05, ** *p *< 0.01

### Knockdown of TRIM52 suppresses CRC cell proliferation

**Fig. 2:**
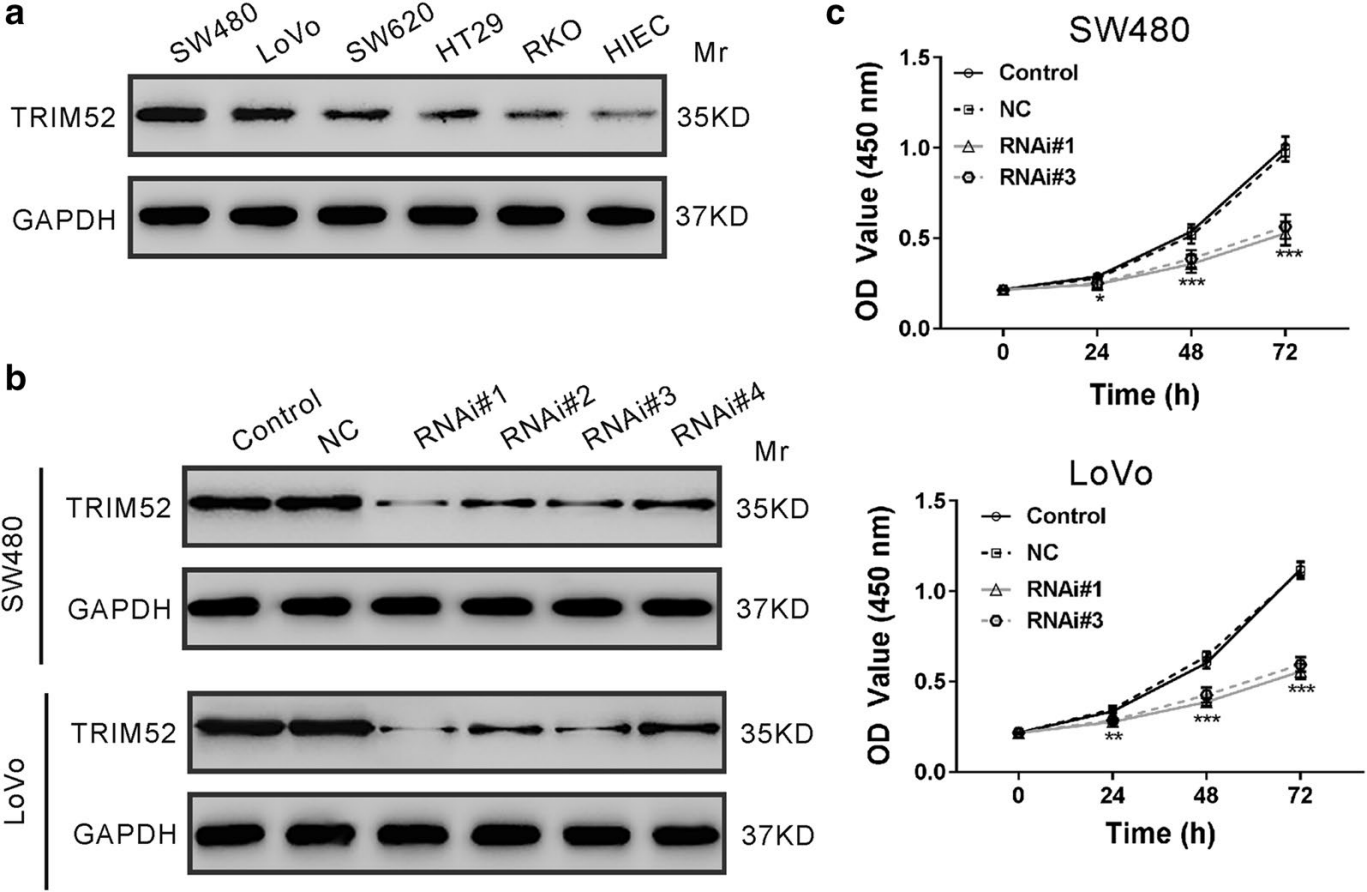
Knockdown of TRIM52 suppresses cell proliferation of CRC cells. **a** Protein expression of TRIM52 in HIEC cell line and 5 CRC cell lines. GAPDH was served as the loading control. **b** SW480 and LoVo cells were transduced with with lentivirus expressing shRNAs against TRIM52 (RNAi#1, #2, #3 or #4) or with control shRNA (NC) for 48 h. TRIM52 protein expression was analyzed by immunoblot assay. Cells without any treatment were served as negative control. **c** CCK-8 assays were performed to assess cell proliferation of SW480 and LoVo cells transduced with indicated virus for 0, 24, 48 or 72 h. *p < 0.05, **p < 0.01, ***p < 0.001 vs. NC cells

TRIM52 protein expression was measured in 5 colon cancer cell lines and normal human intestinal crypt cells (HIEC). Compared to HIEC cells, CRC cell lines showed notably increased expression of TRIM52 especially in SW480 and LoVo cells (Fig. 2a). To discover whether TRIM52 affected the development of CRC, SW480 and LoVo cells were transduced with lentivirus expressing shRNAs against TRIM52 (RNAi#1, #2, #3 or #4) to knock down TRIM52 expression. As illustrated in Fig. 2b, TRIM52 protein levels were obviously reduced in both cell lines transduced with TRIM52 shRNAs in comparison to that without any treatment (Control) or with control shRNA (NC). RNAi#1 and RNAi#3 had better knockdown efficiency and were used in the subsequent experiments. CCK-8 assays demonstrated that the proliferation of SW480 cells were significantly reduced at 24 h, 48 h and 72 h after RNAi#1 and RNAi#3 treatment compared with NC cells (Fig. 2c). The inhibitory ratios were 15.1%, 33.2%, and 47.4% for RNAi#1, and 12.7%, 29.8% and 44.7% for RNAi#3. Similar results were observed in LoVo cells.

### Down-regulation of TRIM52 enhances CRC cell apoptosis

**Fig. 3:**
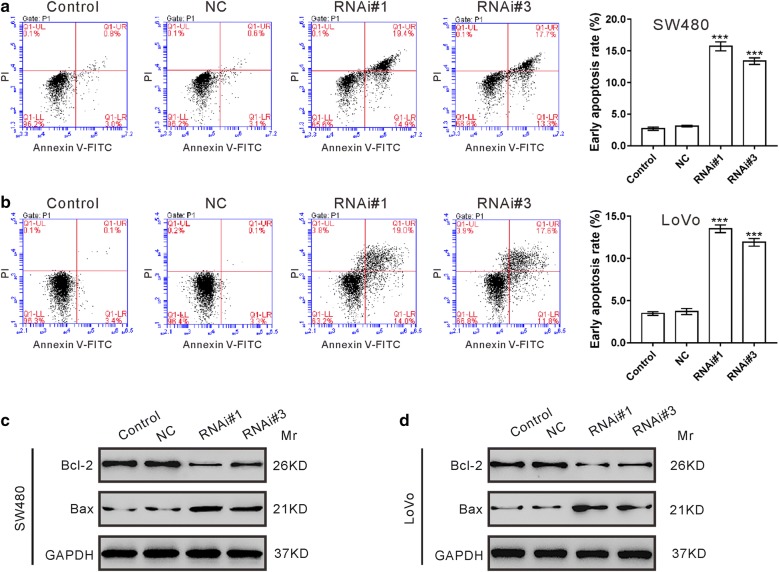
Down-regulation of TRIM52 enhances CRC cell apoptosis. **a**, **b** Annexin V-PI staining followed by cytometry analysis was performed to evaluate cell apoptosis of SW480 (**a**) and LoVo (**b**) cells transduced with indicated virus for 48 h. ***p < 0.001 vs. NC cells. **c**, **d** The protein levels of Bcl-2 and Bax in SW480 (**c**) and LoVo cells (**d**) transduced with indicated virus for 48 h were analyzed by immunoblot assay

To examine whether TRIM52 affected the apoptosis of CRC cells, CRC cells were transduced with RNAi#1, RNAi#2 or NC, cultured for 48 h and then stained with Annexin V-PI and analyzed by a flow cytometer. The apoptosis of SW480 cells (Fig. 3a; apoptotic ratios for Control, NC, RNAi#1 and RNAi#2 were 2.70 ± 0.26%, 3.20 ± 0.10%, 15.70 ± 0.70% and 13.37 ± 0.50%, respectively) and LoVo cells (Fig. 3b; apoptotic ratios for Control, NC, RNAi#1 and RNAi#2 were 3.47 ± 0.21%, 3.70 ± 0.36%, 13.50 ± 0.46% and 11.90 ± 0.46%, respectively) were significantly enhanced by TRIM52 knocking down.

To further verify the pro-apoptosis role of TRIM52 shRNA in CRC cells, the protein levels of apoptosis-related proteins were evaluated by immunoblotting. TRIM52 knockdown caused a significant decrease of anti-apoptotic protein, Bcl-2, and a notable increase of pro-apoptosis protein, Bax in both SW480 (Fig. 3c) and LoVo cells (Fig. 3d).

### TRIM52 shRNA inhibits cell proliferation and induces cell apoptosis in vivo

**Fig. 4:**
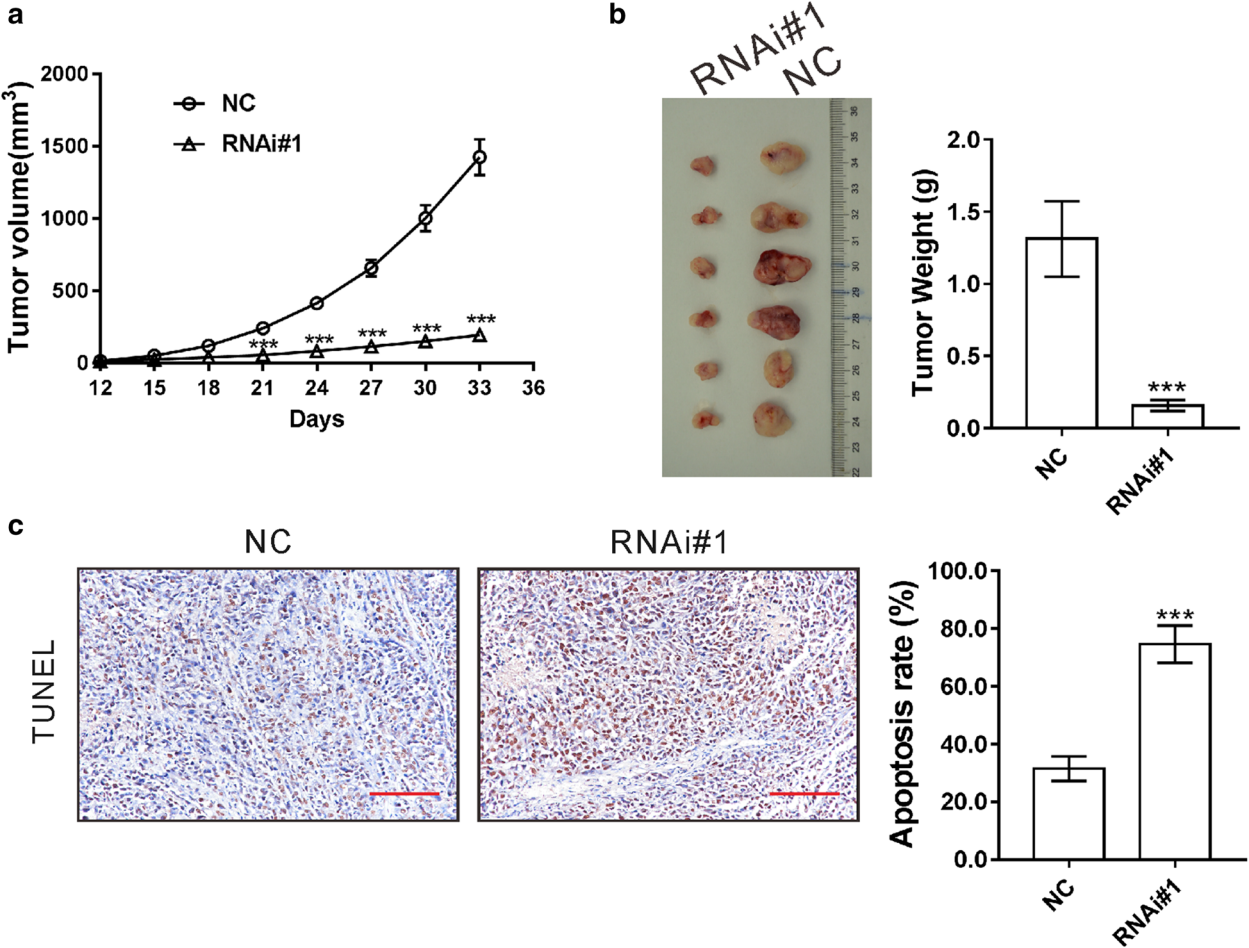
TRIM52 shRNA inhibits cell proliferation and induces cell apoptosis in vivo. LoVo cells stably expressing TRIM52 shRNA (RNAi#1) or control shRNA (NC) were inoculated into nude mice (n = 6). **a** Tumor volume was measured for 33 days. **b**, **c** On Day 33 after cell inoculation, mice were sacrificed, and tumors were resected and weighted. **c** TUNEL assay on the xenograft tumors to evaluate cell apoptosis rate. Scale bar: 100 μm. ***p < 0.001 vs. NC

To determine the effects of TRIM52 knockdown in vivo, LoVo cells stably expressing NC or RNAi#1 were inoculated into nude mice. As shown in Fig. 4a, the xenograft formed by LoVo cells expressing RNAi#1 grew much slower than that by LoVo cells expressing NC in mice. On Day 33 after cell inoculation, the weight of the xenograft formed by RNAi#1 cells was significantly lighter than that of NC cells (Fig. 4b), which suggested that knockdown of TRIM52 could inhibit tumor growth in nude mice. TUNEL assay on the xenograft tumors showed that the apoptosis rate of RNAi#1 cells was 42.2% of that of NC cells (Fig. 4c), indicating that the reduced tumorigenicity was associated with increased cell apoptosis.

### TRIM52 influences CRC cells via modulating the STAT3 signaling pathway

**Fig. 5:**
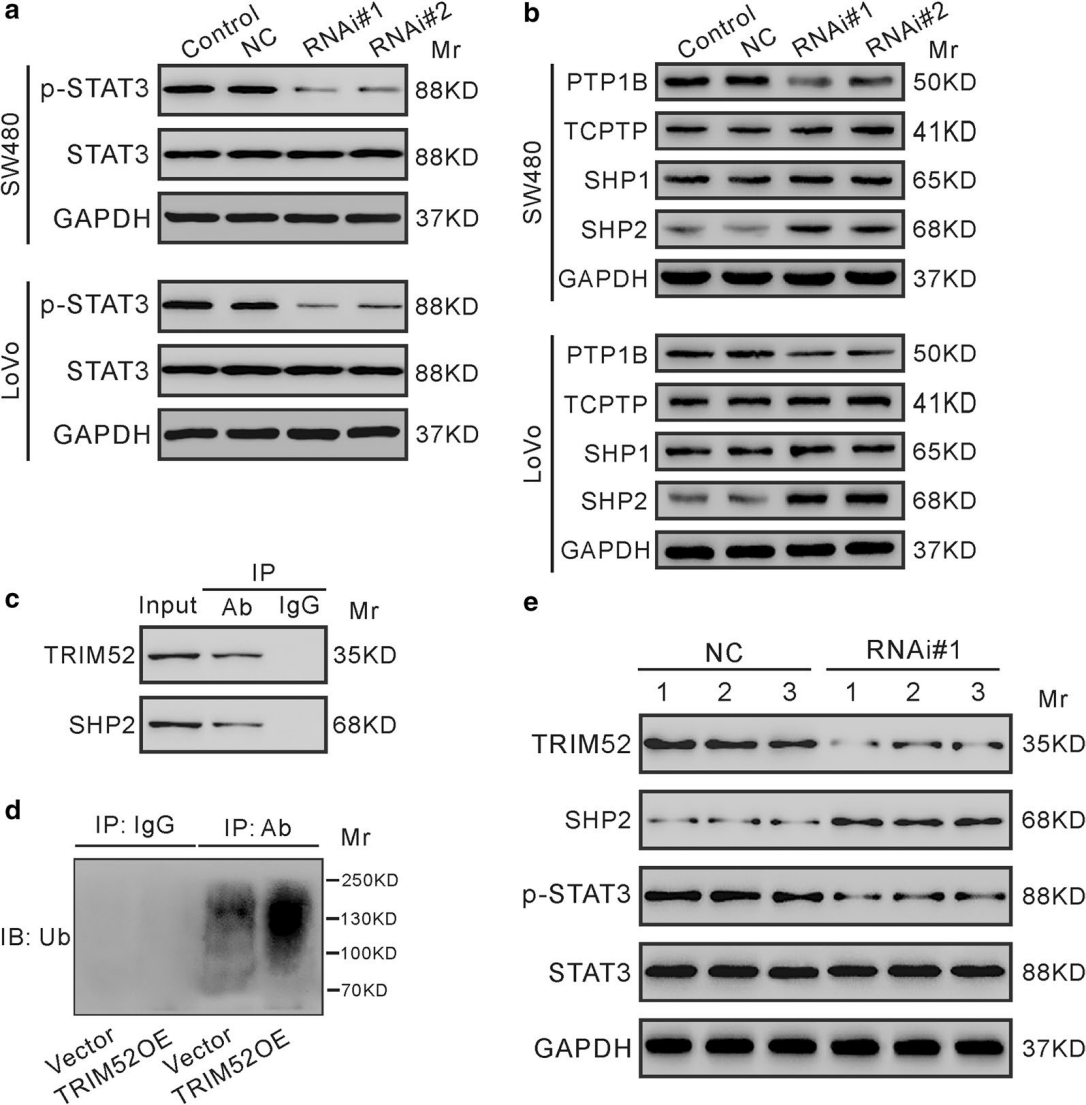
TRIM52 modulates the STAT3 signaling pathway. **a** The levels of p-STAT3 and STAT3 in SW480 and LoVo cells transduced with indicated virus for 48 h were analyzed by immunoblot assay. **b** The levels of PTP1B, TCPTP, SHP1 and SHP2 in SW480 and LoVo cells transduced with indicated virus for 48 h. **c** SW480 cell lysates were immunoprecipitated (IP) with anti-SHP2 (Ab) or immunoglobulin G (IgG), and immunoblotted (IB) with anti-TRIM52 or anti-SHP2. Input: total cell lysate. **d** SW480 cells were transduced with TRIM52 expression lentivirus (TRIM52OE) or control Vector lentivirus. Cell lysates were IP with anti-SHP2 (Ab) or IgG, and IB with anti-Ubiquitin (Ub). **e** The levels of TRIM52, SHP2, p-STAT3 and STAT3 in xenograft tumors were analyzed by immunoblot assay

The STAT3 signaling pathway is known to regulate cell proliferation, survival [20] and differentiation [21]. To examine the effect of TRIM52 on the STAT3 signaling pathway, the levels of p-STAT3 and STAT3 were assessed by immunoblotting. The results revealed the decrease of phosphorylated STAT3 at Tyr705 (p-STAT3) following TRIM52 knockdown in CRC cells (Fig. 5a).

Previous studies have shown that the STAT3 signaling is negatively regulated by protein tyrosine phosphatases (PTPs), including PTP1B [22], TCPTP [23, 24], SHP1 [25, 26] and SHP2 [27]. Thus, we detected the effects of TRIM52 on the protein expression of these PTPs. As illustrated in Fig. 5b, TRIM52 knockdown in CRC cells led to an obvious elevation in SHP2 expression, but had little effect on other PTPs. As TRIM52 possesses E3 ubiquitin ligase activity [16], we then investigate the role of TRIM52 in regulating SHP2 ubiquitination. First, Co-IP experiments with lysates from SW480 cells showed that endogenous TRIM52 and SHP2 were in the same complexes (Fig. 5c). Second, we overexpressed SW480 cells with TRIM52 and observed that TRIM52 overexpression obviously enhanced the polyubiquitination of SHP2 (Fig. 5d), suggesting that TRIM52 may serve as an E3 ligase for SHP2. The effect of TRIM52 knockdown on p-STAT3 and SHP2 was observed also in vivo (Fig. 5e).

**Fig. 6:**
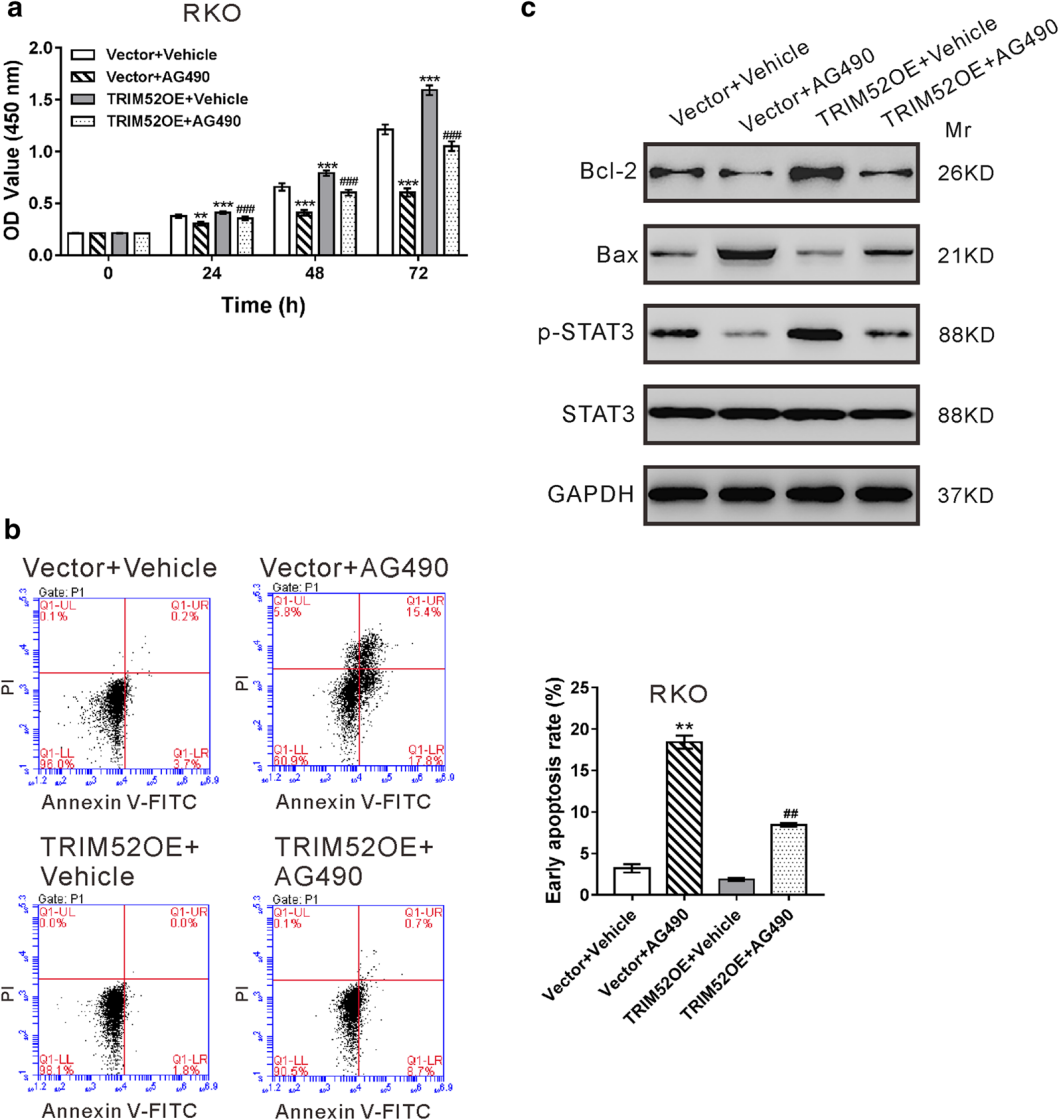
TRIM52 promotes cell proliferation and suppresses cell apoptosis via the STAT3 signaling. RKO cells were transduced with TRIM52 expression lentivirus (TRIM52OE) or control Vector lentivirus in the presence of 100 μM AG490 (Selleck Chemicals, Houston, TX, USA) or vehicle (DMSO). **a** Cell proliferation was detected by CCK-8 assays at 0, 24, 48 or 72 h after treatment. **b** Cell apoptosis was analyzed at 48 h after treatment. **c** The levels of Bcl-2, Bax, p-STAT3 and STAT3 were analyzed by immunoblot at 48 h post treatment. **p < 0.01, ***p < 0.001 vs. cells treated with Vector and vehicle; ^##^*p *< 0.01, ^###^*p *< 0.001 vs. cells treated with TRIM52OE and vehicle

Subsequently, we detected whether JAK inhibitor AG490 could abolish the malignant phenotypes caused by TRIM52 overexpression. As shown Additional file 1: Figure S1, TRIM52 overexpression virus obviously enhanced its protein level in RKO cells, which had a relative lower expression of TRIM52 (Fig. 2a). CCK-8 assay demonstrated that the inhibition of the STAT3 signaling pathway by AG490 repressed the proliferation (Fig. 6a) and enhanced the apoptosis (Fig. 6b) of TRIM52-overexpressed RKO cells. Furthermore, Bax level increased, while the levels of Bcl-2 and p-STAT3 decreased with the inactivation of the STAT3 signaling pathway (Fig. 6c). These data indicated that TRIM52 exerted biological effect through the STAT3 signaling pathway.

## Discussion

The dysfunction of TRIM family proteins implicates in the pathogenesis of various human cancers [12–14]. Recently, studies have investigated the functions of TRIM proteins in CRC. TRIM24 [28] and TRIM29 [29] are up-regulated in CRC tissues and significantly correlated with poor prognosis. Studies have demonstrated the oncogenic roles of TRIM24 [30], TRIM27 [31], TRIM29 [29, 32] and TRIM59 [28] in CRC. TRIM52, a novel member of TRIM family proteins, has been reported to be up-regulated in HCC and to promote cell proliferation, migration and invasion of HCC cells [18, 19]. Nevertheless, the roles and mechanisms of TRIM52 in colorectal carcinogenesis have not been investigated. In present study, we provided new evidence that TRIM52 expression was elevated in CRC tissues (Fig. 1a) and cell lines (Fig. 2a), and that TRIM52 expression was significantly correlated with tumor size, tumor stage and overall survival of CRC patients (Fig. 1b and Table 2). Furthermore, knockdown of TRIM52 expression in CRC cells significantly suppress cell proliferation due to the induction of apoptosis in vitro (Figs. 2, 3) and in nude mice (Fig. 4). Evidence suggested that cellular senescence is another key event contributing to the anticancer response [33]. The possible effect of TRIM52 knockdown on cell senescence could not be ruled out and further work will be necessary. Nevertheless, all of these findings indicate that TRIM52 may serve as an oncogene in CRC, which was consistent with its role in HCC [18, 19].

Activated STAT3 is found to be increased in CRC samples compared to normal mucosae [7–9]. AG490 and STAT3 knockdown could suppress CRC cell growth and invasion, and induce CRC cell apoptosis [9]. A recent study reported that TRIM8 interacts with protein inhibitor of activated STAT3 (PIAS3), thus enhancing the STAT3-dependent signaling [34]. TRIM29 knockdown in CRC cells led to a notable reduce in the phosphorylation levels of STAT3 [32]. Here, we tried to explore the association of TRIM52 and the STAT3 signaling in CRC. TRIM52 knockdown reduced the phosphorylation of STAT3 at Tyr705 in both CRC cells and xenograft tumors (Fig. 5). SHP2, a tyrosine phosphatase, has been shown to negatively regulate the STAT3 signaling [27]. TRIM52 interacted with SHP2 and promotes its ubiquitination, whereas the ubiquitination site on SHP2 is to be identified. Further, we found that JAK2 inhibitor AG490 can block the promotional effects of TRIM52 overexpression on CRC cell proliferation (Fig. 6). These data suggest that TRIM52 may promote SHP2 ubiquitination, thus inactive the STAT3 signaling and serve as an oncogene in CRC.

Bcl-2 family proteins, including anti-apoptotic factor Bcl-2 and proapoptotic factor Bax, are important for the regulation of apoptosis. It seems that abnormal activation of the bcl-2 gene is an early event in colorectal tumorigenesis [35]. The protein levels of Bcl-2 [36] and Bax [37] may be potential prognostic indicator for CRC although controversial results exist. Inhibition of the STAT3 pathway could decrease the Bcl-2 expression and increase Bax expression in CRC cells [9, 38]. Here, TRIM52 knockdown decreased Bcl-2 expression and increase Bax expression (Fig. 3), which may due to the decreased phosphorylation of STAT3. Complementary data were obtained in TRIM52 overexpressed cells (Fig. 6c). The increased apoptosis rate of CRC cells with TRIM52 knockdown may ascribed to the decreased ratio of Bcl2/Bax.

## Conclusion

Our data also revealed that TRIM52 could promote CRC cell proliferation by inhibiting cell apoptosis through the STAT3 signaling pathway. Collectively, these findings provide new insight into the role of TRIM52 in CRC, which might serve as a prognostic indicator and a novel therapeutic target for CRC treatment.

## Additional file

**Additional file 1.** Additional table and figure.

## Abbreviations

CRC: colorectal cancer
STAT3: signal transducer and activator of transcription 3
p-STAT3: phospho-STAT3
siRNA: small interfering RNA
TRIM: tripartite motif
NS2A: nonstructural protein 2A
JEV: Japanese encephalitis virus
HCC: hepatocellular carcinoma
HBX: hepatitis B virus X protein
IHC: immunohistochemistry
shRNAs: short hairpin RNAs
RIPA: radio-immunoprecipitation assay
SDS-PAGE: sodium dodecyl sulfatepolyacrylamide gel electrophoresis
TBST: tris-buffered saline tween-20
GAPDH: glyceraldehyde-3-phosphate dehydrogenase
CCK-8: Cell Counting Kit-8
OD: optical density
SPF: specific pathogen free
TUNEL: terminal deoxynucleotidyl transferase [TdT]-mediated deoxyuridine triphosphate (dUTP)-nick end labelling
ANOVA: one-way analysis of variance
HIEC: human intestinal crypt cells
