# Supplementary material for: TRIM52 promotes colorectal cancer cell proliferation through the STAT3 signaling
Source: Cancer Cell Int. 2019 Mar 14;19:57. doi: 10.1186/s12935-019-0775-4 (PMC6419475; doi:10.1186/s12935-019-0775-4)
Supplement: Supplementary file 1 — Additional file 1. Additional table and figure. [file 12935_2019_775_MOESM1_ESM.docx]

**Table SI.** Interfering RNA (siRNA) sequences for TRIM52.

| **Groups** | **Sequences** |
| --- | --- |
| RNAi#1 | 5’- CCATCTGCTTGGATTACTT -3’ |
| RNAi#2 | 5’-GCTTTACACGTCGCAGCTT-3’ |
| RNAi#3 | 5’- GCATGTGCTTTAAACACCA-3’ |
| RNAi#4 | 5’-CCAGGAAATAAAGTTGGAA-3’ |
| NC | 5’-CCTAAGGTTAAGTCGCCCTCG-3’ |


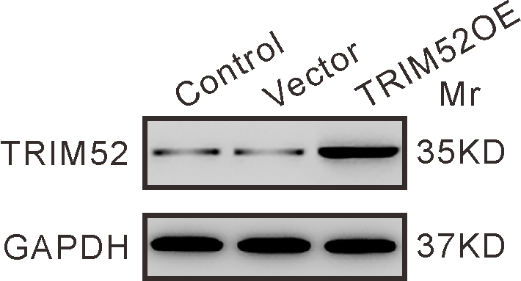


**Figure S1.** The protein levels of TRIM52 in RKO cells after transfected with TRIM52 overexpression virus (TRIM52OE) and control Vector virus.
